# Association between Bone Lead Concentration and Aggression in Youth from a Sub-Cohort of the Birth to Twenty Cohort

**DOI:** 10.3390/ijerph19042200

**Published:** 2022-02-15

**Authors:** Nonhlanhla Tlotleng, Nisha Naicker, Angela Mathee, Andrew C. Todd, Palesa Nkomo, Shane A. Norris

**Affiliations:** 1 Epidemiology and Surveillance Section, National Health Laboratory Service, National Institute for Occupational Health, Braamfontein, Johannesburg 2001, South Africa; nnaicker@uj.ac.za; 2 Environmental Health Department, Faculty of Health Sciences, University of Johannesburg, Johannesburg 2028, South Africa; angie.mathee@mrc.ac.za; 3 Faculty of Health Sciences, School of Public Health, University of the Witwatersrand, Johannesburg 2193, South Africa; 4 Environment and Health Research Unit, South African Medical Research Council, Johannesburg 2094, South Africa; 5 Department of Environmental Medicine & Public Health, Icahn School of Medicine at Mount Sinai, New York, NY 10029, USA; andrew.todd@mssm.edu; 6 SAMRC Development Pathways for Health Research Unit (DPHRU), University of the Witwatersrand, Johannesburg 5050, South Africa; palesa.serendipitycards@gmail.com (P.N.); shane.norris@wits.ac.za (S.A.N.); 7 Global Health Research Institute, School of Health and Human Development, University of Southampton, Southampton SO17 1BJ, UK

**Keywords:** bone lead, blood lead, aggression, BT20 cohort, KXRF, late adolescence, South Africa

## Abstract

Background: An association between blood-lead levels and aggression has been demonstrated in children and adolescent youth in South Africa. However, there are limited studies that have assessed aggression as an outcome for cumulative lead exposure using bone lead concentration. This study aims to assess the association between bone lead concentration and aggressive behaviour among a sample of youth in South Africa. Methods: Bone lead in 100 participants (53 males and 47 females) recruited and followed in the Birth to Twenty (BT20) Cohort were measured using 109 Cd-based, K-shell X-ray fluorescence (KXRF). The Buss–Perry Aggression questionnaire was used to measure aggressive behaviour. Linear regression models were fitted to determine the association between aggression score for physical, verbal, anger and hostility and bone lead, adjusting for known confounders. Results: A one-microgram-per-gram increase in bone lead was found to increase the score for all four scales of aggression, but significantly only for anger (β = 0.2 [95% CI 0.04–0.370]). Psychosocial factors such as a history of family violence and exposure to neighbourhood crime were significant predictors for aggression. Conclusions: The study provides a preliminary overview of the relationship between cumulative lead exposure and behavioural problems such as aggression. A larger sample, across exposed communities, may prove more definitive in further investigating the association between these two important public health factors and to maximize generalizability.

## 1. Introduction

Lead exposure has been found to have a significant impact on the health of exposed children [1,2]. Children who have been exposed to lead have been found to present an elevated risk of delinquent behaviour during adulthood and later adulthood [3]. At low exposure levels, lead may cause acute and long-term health effects, such as neurotoxicity, that can subsequently affect behaviour and compromise intellectual abilities [3,4]. Environmental pollution, contaminated soil, dust, water and food, as well as painted children toys, are sources by which the public becomes exposed to lead [3]. As an example, lead is released into the environment from deteriorating lead paint applied to walls, door frames and windows in houses, schools and other buildings [5,6].

The Port Pirie Cohort study in Australia was one of the first studies to evaluate the association of elevated blood lead levels (BLLs) and “emotional and behaviour problems” in children [7]. In the study, a significant association between behavioural problems, including aggressive behaviour and cumulative BLLs, was found in boys and girls ages eleven to thirteen years [7]. In South African youth, elevated blood levels were significantly associated with aggressive behaviour in young boys (11–13 years), after adjusting for socioeconomic status (SES) factors [8,9]. Similarly, Nkomo et al. (2018) reported an association between elevated BLLs in adolescents aged fourteen to fifteen years and direct forms of aggression, such as verbal and physical [10].

Studies have also investigated the association between bone lead concentration, a measure of cumulative lead exposure, and behavioural problems, including aggressive behaviour: Needleman et al. (1996) reported a significant association between elevated bone lead concentration and aggression in eleven-year-old American boys [11]. In a subsequent study, Needleman et al. (2002) assessed the association between bone lead levels and criminal behaviour in convicted delinquents, aged 12–18 years [12]. In the study, bone lead levels in detained, delinquent youth were reported to be higher (mean 11, SD ±33 ppm) than those in a control group (mean 1.5, SD ± 32 ppm) of delinquent high-school youths. The odds of delinquent behaviour were shown to be greater in youth with higher bone lead levels compared to those with lower levels [12].

One study in 2009, conducted in South Africa by the Centre for Justice and Crime and other related studies identified some of the key factors that may heighten aggressive behaviour in both young males and females [13,14]. These included psychosocial factors such as a history of family violence, exposure to crime and violence, interaction with delinquent peers and substance abuse and the home environment situation [9,10,15]. It has been shown that children raised in households with single parents may show tendencies of aggressive behaviour compared to those who were raised in households with two parents [15]. In addition, exposure to lead can be exacerbated by social determinant factors, such as poverty, low level of education and poor living conditions [16,17,18,19]. While studies assessing lead exposure, aggressive behaviour and delinquency have mainly focused on children (age 14 to15 years), this study assessed the association between bone lead levels and aggressive behaviour among males and females in early adulthood (age 23 to 24 years), followed within a cohort of children previously exposed to lead. Furthermore, our study uses structural equation modelling (SEM) to assess direct and indirect pathways in the relationship between bone lead concentration and aggression. Identifying environmental risk factors that may increase aggressive behaviour among the youth may assist in determining preventable factors associated with aggression.

## 2. Materials and Methods

### 2.1. Study Setting/Site

The BT20 Cohort was established in South Africa (SA) in 1988 with the aim of conducting a longitudinal study to assess the health of children in the Johannesburg area [20,21]. The study enrolled women in their second and third trimester of pregnancy. Singleton children (*n* = 3273) who were residents of Soweto-Johannesburg and born between April and June 1990 were enrolled into the birth cohort and were followed from birth. Approximately 2300 children and their families were still participating in the study by 2018. The entry inclusion criterion into the cohort was that mother and baby remain in the Soweto-Johannesburg area until the child was at least six months old. The attrition rate in the first two decades of the cohort was low (30%), with most occurring in infancy and early childhood. 

### 2.2. Study Population 

A sub-cohort (approximately *n =* 500) from the birth cohort study was formed at age 9 years to investigate the in-depth longitudinal changes in body composition and whole-body, lumbar spine and hip-bone mass during adolescence and into adulthood (Bone Health Cohort). The current study sample was comprised of young adult (23 and 24 years) males (*n =* 53) and females (*n =* 47) recruited from Bone Health Cohort in Johannesburg, South Africa. 

### 2.3. Bone Lead Measurements

Bone lead concentration was the exposure variable in this study. Tibia-lead concentration was measured using K-shell X-ray fluorescence (KXRF), a non-invasive procedure [22]. Tibia bone located in the lower leg was selected as a measurement site as it is one of the larger cortical bones in the body with more bone mass and a minimal amount of overlying soft tissue than trabecular bone structures allowing for maximum excitation of bone tissue by the photon beam [23,24]. Results from KXRF spectrometry of tibial bone in humans have been used for decades in dozens of studies as a biomarker to assess cumulative lead exposure levels [25,26,27,28]. XRF uses ^109^Cd as the source that emits 88.035 keV photons to fluoresce X-rays from the lead atoms stored in bone. The silver X-rays that also accompany the decay of ^109^Cd are filtered by copper, minimizing participant radiation dose (an effective dose is equivalent to less than ten minutes of natural background radiation for an adult). Backscattered photons and fluoresced Pb X-rays are recorded with a spectroscopy system (intrinsic germanium detector, preamplifier and digital signal processor). The spectrum (distribution of photons against energy) then undergoes non-linear least-squares fitting to extract the areas of the lead X-ray peaks seen atop the Compton scattering background. Coherent scatter normalization, matrix correction and comparison to calibration measurements, made of lead-doped plaster of Paris (CaSO_4_·2H_2_O) calibration standards, yield in vivo concentrations in micrograms of lead per gram of bone mineral. Measurements were repeated for accuracy, where bone levels outside the calibration curve were removed from the analysis.

### 2.4. Measurement of Aggression 

Aggression was the outcome variable in the study. The Buss–Perry Aggression Questionnaire (BPAQ) was administered to study participants to measure aggression as a score [29]. The BPAQ is a validated tool that has been used in studies primarily in low- to middle-income countries [30,31]. The questionnaire consists of twenty-nine items that measure four components of aggression: physical and verbal aggression, hostility and anger. Physical aggression consisted of nine questions, and the scoring from this item ranged from 18 to 38. Verbal aggression had five items, with the scoring ranging from 10 to 25. Anger consisted of seven items, with a scoring range of 14 to 35; and hostility consisted of eight items, with a scoring range of 10 to 37 [31]. The level of aggression in the questionnaire was rated on a five-point Likert scale, presented as 1 (extremely uncharacteristic of me), 2 (somewhat uncharacteristic of me), 3 (neither uncharacteristic nor characteristic of me), 4 (somewhat characteristic of me) and 5 (extremely characteristic of me). The total aggression scores of the twenty-nine items were also calculated, and these were also used in the analyses. A Cronbach’s α reliability coefficient was used to determine the reliability of the items. An alpha coefficient of 0.72 was obtained for the nine items of physical aggression, 0.6684 for the seven items of anger, 0.7150 for the eight items of hostility and 0.5640 for the five items of verbal aggression, indicating acceptable reliability among the items. The 29 items reported a scale reliability coefficient of 0.8364, similar to that reported previously [29]. 

### 2.5. Study Confounders 

A separate questionnaire was administered to the study participants to obtain information on demographics, socio-economic and psychosocial factors. A confounding variable in the study was defined as a variable that is a risk factor for aggression or is associated with, but is not a consequence of, bone lead concentration. The following variables were considered as study confounders and potential predictors of aggression: age; sex; level of schooling (categorized into three: Grade 5 or less, grade 6–12 and tertiary education); presence of both parents at home; home environment; neighbourhood crime; profile of illegal substance abuse (use of drugs such as cannabis commonly known in Southern Africa as “dagga” and glue); use of alcohol; and socio-economic factors (maternal education, type of housing and occupation status). Information on the participant’s home environment (was referred to in this analysis as “history of family violence”) was obtained by asking the participants to respond to the following statements: “We argue a lot in our family, “people in my family hardly ever lose their temper” and “people in my family sometimes hit each other when they are angry”. Participant were required to agreed or responded with a “Yes” (coded as 1) or disagreed or responded with a “No” (coded as 2). To obtain information on neighbourhood factors, the participants were asked how they generally feel in their neighbourhood: a “feeling of somewhat unsafe or very unsafe” coded as 1 and “somewhat safe or very safe” coded as 2. Questions on whether the participants had personally experienced crime and violence in the neighbourhood were asked: “ever in your life experienced any crime”, with “Yes” coded as 1 and “No” coded as 2. Socioeconomic status was measured by considering three levels: (1) maternal level of education (categorized into four levels: no formal education, primary schooling, secondary schooling and post-school education); (2) type of housing: formal (such as a free-standing house, townhouse or hostel) and informal (shack, squat and any other informal room); and (3) participant’s level of education and occupational status as proxies. 

### 2.6. Ethical Consideration

Ethical approval was obtained for this study from the University of the Witwatersrand, Human Ethics Research Committee (M 191116).

### 2.7. Statistical Analysis 

All data cleaning and analysis were performed using Stata version 15 (StataCorp. 2017; College Station, TX, USA). Data were checked for duplicates and missing values. Study participants’ psychosocial and demographic characteristics were described. The categorical variables were presented as frequencies and proportions. Data were stratified by sex. The Pearson chi-squared test was used to assess the association between categorical variables. Continuous variables in the study were bone lead concentration and the four aggression level scores (physical, verbal, anger and hostility). Tibia lead concentrations were analysed in the study as a continuous variable to retain all values, including values below the detection limit and values lower than zero. The distribution of the continuous variables was checked for normality. Bone lead concentrations were summarized as mean and SD, median, 25th and 75th Interquartile ranges (IQR), as appropriate. For continuous variables that were normally distributed, an independent Student’s *t*-test was conducted to test for the difference in the mean. There was no further post hoc correction analysis required. Testing was set at the 0.05 level of significance. The geometric mean, median and ranges for the aggression scales were described, and a Student’s *t*-test was conducted to test for the differences in means, stratified by sex. 

To assess the association between bone lead concentration and aggression, a linear regression model was fitted. In the univariate analysis, a simple linear regression was fitted, with each aggression scale as the outcome variable and with bone lead as the main explanatory variable. A backward elimination, using a liberal *p*-value of 0.20, was used to include variables in the multivariate model. Variables with *p ≤* 0.001 were reported as highly significant, and those with *p ≤* 0.08, also retained in the final model, were reported as marginally significant. Age and sex were retained as study confounders in all multivariable models. Goodness of fit was assessed via regression diagnostics, and residuals were assessed to check the assumptions of linearity, normality and constant variance and the adequacy of the final models. 

To further quantify and assess the direction of the relationship between bone lead levels and aggression, structural equation modelling (SEM) was performed. In the SEM model, aggression was the latent variable, which, as previously indicated, was assessed via four observed variables: physical aggression, verbal aggression, hostility and anger. All observed variables were denoted by rectangular boxes, and latent variables (unobserved) were denoted in ovals. In model I, pathways between educational level, age, sex, type of housing, occupational status and maternal education were created, as these variables were identified as determinants of bone lead levels [27,32]. Subsequently, the variables (educational level, age, sex, type of housing, occupational status and maternal education) created indirect pathways to aggression via (continuous) bone lead concentration. Direct predictors for aggression in model II were: a history of family violence, exposure to crime, growing up with a single parent and use of drugs and alcohol. To assess model fit, SEM fit indices that included: the root mean square error (RMSE), standardized root mean square residual (SRMR), comparative fit index (CFI) and Tucker–Lewis index (TLI). An RMSE below 0.05, P-close greater than 0.05, SRMR greater than 0.08, CFI and TLI value of 0.95 and above indicated a good fitting model [33,34]. Where necessary, the model was checked for improvement using modification of indices.

## 3. Results

The results of the socio-demographic and psychosocial characteristics of the study sample stratified by sex are shown in Table 1. Overall, the sample consisted of 53 male and 47 female participants. Close to 60% of the study participants reported living with a single parent; 58.7% (*n =* 27) of females and 62.3% (*n =* 33) of males. Most of the study participants had a secondary-level education: 60.4% (*n =* 32) of the males and 61.7% (*n =* 29) of the females; only a few (10%) had tertiary/post-school education. For maternal education, a large proportion of participants (78%) reported their mothers to have had secondary-level education. 

Only 3% of the study participants reported living in informal dwellings such as a shack. Approximately half the participants were employed on a casual/part-time basis or self-employed (52%). Experiences in the home environment were obtained by asking whether the participants had “ever” experienced violence or aggressive behaviour in the family, and most (67%) disagreed with the statement. In addition, many of the participants reported a feeling of being unsafe or somewhat unsafe in their neighbourhood: 84.9% (*n =* 45) of males and 74.5% (*n =* 35) of the females. Previous use of alcohol and drugs was more common among males than in females, with 100% of males (*n =* 53) reporting having used alcohol and 75.5% (*n =* 40) of males reporting having used illicit drugs such as hallucinogens, cannabis, cocaine, inhalants and opiates, among others. A chi-square test showed the use of drugs and alcohol to be significantly different between males and females (*p* < 0.001). 

The concentration of bone lead in males and females is summarized in Table 2. Bone lead levels in females were marginally greater than those of males, but this difference was not statistically significant. The range was 5–11 µg/g bone mineral in males and 4–14 µg/g in females.

The geometric mean, median and ranges of the four scales of aggression, stratified by sex, are shown in Table 3. Higher scores indicate more aggressive behaviour. Males reported an insignificant greater mean score for physical aggression (27.2 and a geometric mean of 26.7) than females. On the contrary, females scored insignificantly greater for anger, hostility and verbal aggression. The total score for aggression was also insignificantly greater in females (93.8) than in males (91.8). 

Bone lead concentration was found not to be significantly associated with physical aggression, verbal aggression and hostility (see supplementary tables) but was significantly associated with anger. Table 4 and Table 5 show the univariate and multivariable analysis of mean aggression score for anger and bone lead concentration, as well as anger and other study predictors. Factors such as maternal education, being exposed to family violence and a feeling of being unsafe in the neighbourhood were significantly associated with anger in the unadjusted model.

In Table 5, the final model shows that previous exposure to family violence, “feeling somewhat unsafe/very unsafe toward neighbourhood” and being exposed to neighbourhood crime and violence were significantly associated with mean aggression score for anger. Adjusting for confounding variables, the linear regression model shows that a one-microgram-per-gram increase in bone lead significantly increases the mean aggressive score for anger by 0.25 [95% CI: 0.04–0.37]. This increase was similar for physical aggression (coefficient = 0.093 [95% CI: −0.01–0.27]); verbal aggression (coefficient = 0.093 [95% CI: −0.05–0.23]) and hostility (coefficient = 0.030 [95% CI −0.19–0.26]) (see Supplementary Tables S1–S3).

**Figure 1:**
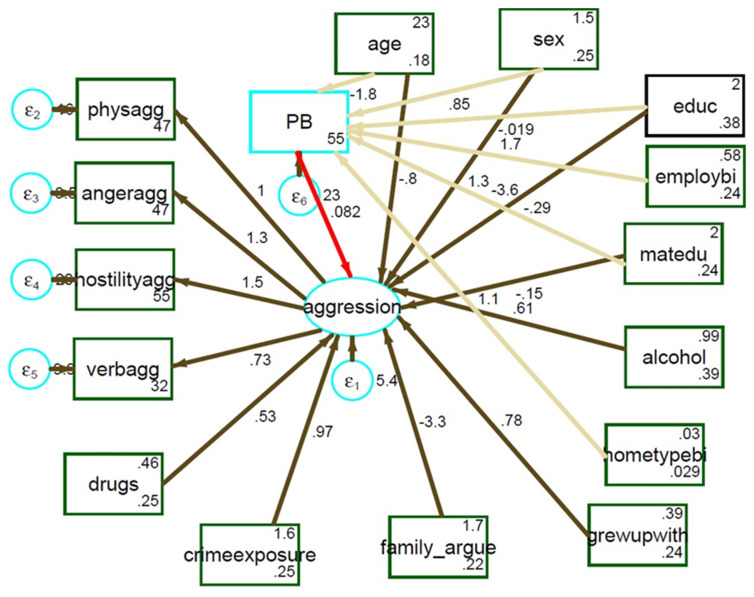
Path analysis between PB, aggression and study predictors. (Fit statistics: χ^2^ (model vs. saturated) = 48.403 (0.337); RMSE = 0.028 (pclose 0.736); CFI = 0.970; TLI = 0.957; SRMR = 0.048 and coefficient of determination = 0.43) (matedu = maternal education; hometypei = type of housing; family argue = exposure to family violence; crimeexposure = crime and violence in neighbourhood; employbi = occupational status).

Figure 1 shows the structural equation model with direct and indirect pathways between PB and aggression and between PB and variables considered to be determinants of lead levels. The indirect pathways to aggression show the effect of socioeconomic status predictors (education, maternal education, and employment status) and demographics (age, sex) on PB (light khaki arrows). Consistent with the linear regression analysis (Supplementary Table S4), a positive path coefficient between PB and aggression (0.082) was obtained in a direct pathway (red arrow), but this pathway was not statistically significant (*p* = 0.183) (pathways to other predictors of aggression are shown in light brown). The pathway from maternal education to PB was found to be significant (*p* < 0.001). The path coefficients between age, maternal education and PB showed negative coefficients. The pathway between history of family violence and aggression was found to be significantly associated with aggression (*p* < 0.001).

## 4. Discussion

This study investigated the association between bone lead levels and aggressive behaviour among late-adolescent youth, aged 23 and 24 years. Since lead has been reported to have a faster turnover rate in trabecular bones than in the cortical bones, tibia bone was selected as the measurement site for bone lead levels in the study population [25,27]. Lead exposure has been reported to cause adverse health effects in minority groups, including changes in behaviour. In this study, the mean bone lead concentration was 8.1 (SD 4.4) µg/g in males and 9.4 (6.1) µg/g in females. Sex has been shown to be an important determinant of bone lead levels [32,35]. Our results showed that the mean concentration of bone lead was slightly higher in females compared to males, even though the difference was not statistically significant, consistent with Roy et al., 1997. 

The description of the psychosocial, demographic and socioeconomic characteristics, stratified by sex, showed that the majority of the participants in the study did not grow up with both their parents (60.6%) and that most reported living in formal housing (97%). As reported in the literature, children who grow up in households with single parents were found to display more aggressive behaviour compared to those who grew up with both parents [15]. Other sociodemographic factors, including low income, education status and dwelling in older housing, have been attributed to higher bone lead levels in children [36,37]. These factors, though found in this study not to be significant in the final model between bone lead concentration and anger, have been reported as risk factors for aggressive behaviour in lead-exposed children [9,10]. 

The analysis showed that the use of illicit drugs and alcohol was more prevalent in males than in females. In a multivariable model, alcohol and drug use were found not to be significant as confounding factors in the association between bone lead and aggression. Illicit drugs are considered sources of lead exposure, where marijuana, methamphetamine and ingestion of homemade opium such as heroin has been reported in incidence of inorganic lead poisoning [38,39]. Varying trends in sex difference in alcohol and certain illicit drug use, such as heroin and hallucinogens, have been seen: although in early and mid-adolescent years, substance use in females matches that of males, in late adolescence the prevalence of substance use tends to become greater in males than in females [14,40]. The mean score for physical aggression among the males in this study was slightly higher than the mean score for females, findings from similar studies: irrespective of age [8,41], males reported more direct forms of aggression, such as physical aggression, than females, whereas females reported indirect forms of aggression such as hostility and anger [16]. A small sex difference has previously been detected for hostility [31]. 

After adjusting for study confounders, we found a significant association between bone lead levels and anger aggression. In addition, the model showed a positive coefficient indicating that an increase in bone lead concentration increases the aggression score for anger. Reports on the psychological impact of anger show that anger affects the brain by compromising the neurons in the hypothalamus where the stress response occurs [42]. In the literature, the adverse effect of lead on brain function has been demonstrated; thus, the accumulation of lead in the body may activate or trigger feelings of anger in individuals. Nonetheless, more detailed explanations of biological mechanisms are needed. Furthermore, it has been shown that individuals who exhibit anger usually have other aggressive behaviours such as hostility [42]. Even though bone lead levels were not associated with physical aggression, hostility and verbal aggression, anger may be the principal way of expressing aggression and, hence, the strong association in lead-exposed individuals. The adjusted analysis between bone lead levels and anger in this study showed that a history of family violence and unsafe neighbourhoods were also associated with anger aggression. Children who grow up in a home environment where there is shouting and violence will tend to be angry at their situation [42].

Lower education levels, including those for maternal education status, are indirect measures of poor socioeconomic status and have been reported to be strongly associated with higher lead levels [43]. SEM analysis showed a significant negative path coefficient between higher maternal education and bone lead concentration, indicating that participants whose maternal parent has attained a higher education level would report lower bone lead levels. Nonetheless, in a direct pathway between maternal education and aggression, we found no association, where higher education levels of the mothers indicated a decrease in aggressive behaviour. In other studies, lower parental education has been associated with aggressive behaviour in children [44].

Disadvantaged communities in poor-resourced countries have been shown to be at a higher risk for environmental lead exposures [45]. Environmental pollution from dry peeling paint from old houses, schools and buildings previously painted with lead-containing paint may be the primary sources of Pb exposure in disadvantaged communities such as the one in this study. In this regard, the possible major route of exposure for these communities could be the inhalation of lead-containing dust. In addition, in early childhood exposure, lead is accumulated and could be released at a later stage due to bone remodelling and growth in later childhood [32]. In this study, we found that the pathway between the type of housing and bone lead levels showed a non-significant association. Considering that the BT20 Plus Cohort is a community study conducted in the south-western township of Johannesburg where the homes are estimated at over 50 years old, it was expected that formal housing likely decorated with leaded paint would show a strong association with bone lead levels. 

### Study Strength and Limitations

One of the strengths of this study is the use of a non-invasive, authoritative and sensitive measurement procedure was used to quantify bone lead levels in the cohort. Research that has used KXRF for repeated measurements of bone lead levels has shown that the instrument provides credible precision of values compared to chemical analyses. Secondly, a reliable and validated tool was employed to quantify aggression scores in the study. Nonetheless, this study may present a limitation in terms of the sample size, making the results not generalizable to the population. A lack of correlation between lead exposure and other environmental heavy metals known to result in long-term neurotoxic effects is a limiting factor of the current study; nonetheless, this is an important factor to be considered for future studies. Other limitations include the potential for bias, such as recall bias where participants may tend to underreport their aggressive behaviour patterns. 

Information on other factors, such as occupational history related to lead exposure, smoking history, housing age or duration of residence at the current house of participants, as well as the physical appearance of the homes in terms of peeling paint, was not collected. These factors could have improved the variance in the model explaining the relationship between bone lead levels and anger aggression. In addition, other risk factors for aggression were not included in the psychosocial questionnaire, such as paternal history and food insecurity. Research has linked poor nutrition, more common in poorer communities, and low-income brackets with an increase in the body’s lead absorption [46]. 

## 5. Conclusions 

In summary, this study showed that a unit increase in cumulative lead exposure increased the mean score of aggression in late adolescents. Bone lead concentrations were significantly associated with the aggression score for anger. This effect might pose adverse effects later in life that include violent behaviour [47] and participation in criminal activities [48]. Further longitudinal studies employing a larger sample size in South African youth are needed to investigate more fully the relationship of these two important public-health factors. 

## Figures and Tables

**Table 1:** Description of socio-demographic and psychosocial characteristics of study participants stratified by sex.

Variable	Males (*n*, %) (*n =* 53)	Females (*n*, %) (*n =* 47)	Total (N = 100)
**Socio-Demographic Factors**			
**Living with Both Parents (missing *n* = 1)**			
Yes	20 (37.7)	19 (41.3)	39 (39.4)
No	33 (62.3)	27 (58.7)	60 (60.6)
**Level of Education**			
Grade 5 or less	14 (26.4)	8 (17.0)	22 (22.0)
Grade 6–12	32 (60.4)	29 (61.7)	61 (61.0)
Tertiary	7 (13.2)	10 (21.3)	17 (17.0)
**Maternal Education**			
No formal education	0	1 (2.1)	1 (1.0)
Primary	5 (9.4)	6 (12.8)	11 (11.0)
Secondary	42 (79.3)	36 (76.6)	78 (78.0)
Post-school training	6 (11.3)	4 (8.5)	10 (10.0)
**Socio-Economic Factors**			
**Type of Housing**			
Formal housing (RDP/hostel/free standing)	51 (96.2)	46 (97.9)	97 (97.0)
Informal (shack)	2 (3.8)	1 (2.1)	3 (3.0)
**Occupation**			
Employed, full time	19 (35.9)	18 (38.3)	37 (37.0)
Casual/part-time/self-employed	28 (52.8)	24 (51.1)	52 (52.0)
Never been employed	6 (11.3)	5 (10.6)	11 (11.0)
**Home and Neighbourhood**			
**Exposure to family violence**			
Yes	19 (35.9)	14 (29.8)	33 (33.0)
No	34 (64.1)	33 (70.2)	67 (67.0)
**Attitude toward Neighbourhood**			
somewhat unsafe/very unsafe	45 (84.9)	35 (74.5)	80 (80.0)
Somewhat safe/very safe	8 (15.1)	12 (25.5)	20 (20.0)
**Exposure to Crime and Violence in Neighbourhood**			
Yes	25 (47.2)	19 (40.4)	44 (44.0)
No	28 (52.8)	28 (59.6)	56 (56.0)
**Substance Abuse**			
**Drugs**			
Yes	40 (75.5)	6 (12.8)	54 (54.0)
No	13 (24.5)	41 (87.2)	46 (46.0)
**Alcohol**			
Yes	53 (100)	42 (89.4)	95 (95.0)
No	0	5 (10.6)	5 (5.0)

**Table 2:** Bone lead concentrations.

Continuous Bone Lead Levels (µg/g Bone Mineral)	Males (*n =* 53)	Females (*n =* 47)	Total N *=* 100	*p*-Value *
Mean (SD)	8.1 (4.4)	9.4 (6.1)	8.7 (5.3)	0.2021
**Range**				
Minimum	0	0	0	
Median (IQR)	8 (5–11)	10 (4–14)	9 (5–12.5)	
Maximum	18	21	21	

* Student *t*-test used to obtain *p*-value.

**Table 3:** Geometric mean, median and range of aggression scores by sex.

	Males (*n* = 53)			Females (*n* = 47)			*p*-Value *
Aggression Scale	Geometric Mean	Mean (SD)	Range	Geometric Mean	Mean (SD)	Range	
Physical aggression	26.7	27.2 (4.7)	18–36	26.0	26.5 (5.1)	18–38	0.4782
Anger	20.5	20.9 (4.4)	14–32	21.7	22.3 (5.3)	14–35	0.1615
Hostility	24.9	25.7 (6.2)	10–38	25.8	26.5 (5.9)	14–37	0.5193
Verbal aggression	17.0	17.4 (3.8)	10–25	18.2	18.6 (3.7)	10–25	0.1180
Total score	90.0	91.2 (14.3)	57–118	92.6	93.8 (15.4)	66–127	0.3768

* Student *t*-test used to obtain *p*-value.

**Table 4:** Unadjusted univariate model showing the association between bone lead concentration and anger aggression.

Factor	Mean	*p*-Value	95% CI	
**Pb**	0.1	0.246	−0.076	0.292
**Age**				
=23 years	baseline (0)			
=24 years	−0.3	0.822	−2.572	2.047
**Sex**				
Male	baseline (0)			
Female	1.4	0.162	−0.558	3.299
**Grew up with Both Parents**				
Yes	baseline (0)			
No	−0.4	0.701	−2.304	1.555
**Education Level**				
Grade 5 or less	2.3	0.157	−0.875	5.329
Grade 6–12	0.1	0.941	−2.537	2.733
Tertiary	baseline (0)			
**Maternal Education**				
No formal education	9.8	0.047 **	0.147	19.366
Primary	2.0	0.193	−1.046	5.104
Secondary	baseline (0)			
Post school training	−0.1	0.929	−3.351	3.063
**Type of Housing**				
Formal	baseline (0)			
Informal	1.8	0.523	−3.850	7.527
**Occupation**				
Employed	baseline (0)			
Casual	1.8	0.084	−0.249	3.891
Unemployed	1.1	0.526	−2.246	4.364
**Exposure to Family Violence**				
Yes	4.3	<0.001 ***	2.459	6.212
No	baseline (0)			
**Attitude toward Neighbourhood**				
somewhat unsafe/very unsafe	−2.6	0.031 **	−4.998	−0.252
Somewhat safe/very safe	baseline (0)			
**Exposure to Crime and Violence (Neighbourhood)**				
Yes	−0.9	0.362	−2.851	1.049
No	baseline (0)			
**Drug use**				
Yes	0.4	0.674	−1.535	2.364
No	baseline (0)			
**Alcohol use**				
Yes	−0.2	0.944	−4.620	4.303
No	baseline (0)			

*** *p* < 0.001 highly significant; ** *p* < 0.05 significant.

**Table 5:** Multivariable model of the association between anger and bone lead, after adjusting for study confounders.

Factor	Mean	*p*-Value	95% CI	
Pb	0.2	0.017 **	0.038	0.370
Age				
=23 years	Baseline (0)			
=24 years	−0.6	0.593	−2.632	1.512
Sex				
Male	Baseline (0)			
Female	1.0	0.231	−0.676	2.765
**Exposure to family violence**				
Yes	4.8	<0.001 ***	2.982	6.691
No	Baseline (0)			
**Attitude toward neighbourhood**				
Somewhat unsafe/very unsafe	−2.3	0.041 **	−4.461	−0.089
Somewhat safe/very safe	Baseline (0)			
**Exposure to crime and violence** **(neighbourhood)**				
Yes	−1.6	0.068 *	−3.383	0.125
No	Baseline (0)			

*** *p* < 0.001 highly significant ** *p* < 0.05 statistically significant * *p* < 0.08 marginally significant.

## Data Availability

The datasets generated and/or analysed during the current study are SAMRC Development Pathways for Health Research Unit (DPHRU), University of the Witwatersrand, Johannesburg, South Africa. The data may be made available on request.

## Supplementary Materials

The following supporting information can be downloaded at: https://www.mdpi.com/article/10.3390/ijerph19042200/s1, Table S1: Assessment of the association between physical aggression, bone lead and individual level confounders: Final model; Table S2: Assessment of the association between verbal aggression, bone lead and individual level confounders: Final model; Table S3: Assessment of the association between hostility, bone lead and individual level confounders: Final model; Table S4: Assessment of the association between total aggression score, bone lead and individual level confounders: Final model.
