# Supplementary material for: Association between Bone Lead Concentration and Aggression in Youth from a Sub-Cohort of the Birth to Twenty Cohort
Source: Int J Environ Res Public Health. 2022 Feb 15;19(4):2200. doi: 10.3390/ijerph19042200 (PMC8871669; doi:10.3390/ijerph19042200)
Supplement: Supplementary file 1 [file ijerph-19-02200-s001.zip › ijerph-1537258-supplementary.pdf]

Table S1. Assessment of the association between physical aggression, bone lead and individual level confounders: Final model

Factors	Mean	p Value	95% CI	
Pb	0.1	0.352	−0.099	0.274
Age				
=23 years	ref			
=24 years	−1.4	0.218	−3.763	0.867
Sex				
Male	ref			
Female	−1.0	0.305	−2.922	0.924
Exposure to family violence				
Yes	2.7	0.012 **	0.606	4.750
No	ref			
Exposure to crime and violence				
Yes	−1.9	0.061*	−3.834	0.086
No	ref			

**$p < 0.05$ significant *$p \leq 0.08$ marginally significant.

Table S2. Assessment of the association between verbal aggression, bone lead and individual level confounders: Final model

Factors	Mean	p Value	95% CI	
Pb	0.1	0.189	−0.046	0.233
Age				
=23 years	ref			
=24 years	0.03	0.967	−1.665	1.735
Sex				
Male	ref			
Female	0.9	0.244	−0.588	2.290
Exposure to family violence				
Yes	1.4	0.069 *	−0.116	2.987
No	ref			
Attitude toward neighbourhood				
somewhat unsafe/very unsafe	−2.8	0.003 **	−4.603	−0.971
Somewhat safe/very safe	ref			

$p < 0.001$ highly significant; **$p < 0.05$ significant; *$p \leq 0.08$ marginally significant

Table S3. Assessment of the association between hostility, bone lead and individual level confounders: Final model

Factors	Mean	P value	95% CI	
Pb	0.03	0.792	−0.196	0.256
Age				
=23 years	ref			
=24 years	−2.9	0.037 **	−5.675	−0.183
Sex				
Male	ref			
Female	0.6	0.638	−1.772	2.878

Exposure to family violence				
Yes	2.8	0.028 **	0.307	5.319
No	ref			
Attitude toward neighbourhood				
somewhat unsafe/very unsafe	−3.7	0.014 **	−6.646	−0.778
Somewhat safe/very safe	ref			

** $p < 0.05$ significant.

Table S4. Assessment of the association between total aggression score, bone lead and individual level confounders: Final model

**Factors**	**Mean**	***p* Value**	**95% CI**	
Pb	0.4	0.108	−0.942	0.934
Age				
=23 years	ref			
=24 years	−5.3	0.103	−11.709	1.086
Sex				
Male	ref			
Female	1.3	0.624	−3.999	6.627
Exposure to family violence				
Yes	11.9	<0.001 ***	6.180	17.632
No	ref			
Attitude toward neighbourhood				
somewhat unsafe/very unsafe	−11.2	0.001 **	−17.979	−4.481
Somewhat safe/very safe	ref			

*** $p < 0.001$ highly significant; ** $p < 0.05$- significant
